# Sequential Fractional CO_2_ and 1540/1570 nm Lasers: A Narrative Review of Preclinical and Clinical Evidence

**DOI:** 10.3390/jcm14113867

**Published:** 2025-05-30

**Authors:** Alessandro Clementi, Giovanni Cannarozzo, Luca Guarino, Elena Zappia, Fortunato Cassalia, Antonio Alma, Mario Sannino, Caterina Longo, Steven Paul Nisticò

**Affiliations:** 1 Department of Dermatology, University of Modena and Reggio Emilia, 41124 Modena, Italy; antonioalma@virgilio.it (A.A.); caterina.longo@unimore.it (C.L.); 2 Department of Clinical Sciences, Sapienza University of Rome, 00185 Rome, Italy; drcannarozzo@gmail.com (G.C.); luca.guarino@uniroma1.it (L.G.); dr.mariosannino@gmail.com (M.S.); steven.nistico@uniroma1.it (S.P.N.); 3 Department of Health Sciences, Magna Graecia University, 88100 Catanzaro, Italy; 4 Dermatology Unit, Department of Medicine (DIMED), University of Padua, 35121 Padua, Italy; fortunato.cassalia@studenti.unipd.it

**Keywords:** fractional CO_2_ laser, non-ablative laser, skin rejuvenation, dual-wavelength laser

## Abstract

Ablative fractional CO_2_ laser is an established tool for dermatologic and aesthetic indications. Non-ablative wavelengths, such as 1540 and 1570 nm, are increasingly being combined with CO_2_ laser to optimise the results while reducing the recovery time. A narrative review of the literature was conducted, including ex vivo, in vivo, and in vitro studies, as well as human clinical trials that evaluated the efficacy, safety, and histological impact of dual-laser systems. Preclinical studies have shown that sequential application of fractional CO_2_ followed by 1540/1570 nm expands the thermal coagulation zone without increasing the ablation depth. At the histological level, the dual protocol promotes collagen remodelling with greater thermal precision. On a clinical level, a combined treatment has shown efficacy in improving scars, striae distensae, skin laxity, and wrinkles, with reduced recovery times compared to CO_2_ monotherapy. Preliminary data also suggest potential benefits in inflammatory conditions such as hidradenitis suppurativa. The sequential CO_2_ + 1540/1570 nm combination represents an effective and well-tolerated approach in regenerative dermatology. Current evidence supports its use as a versatile, safe, and reproducible technique for skin rejuvenation and scar modulation; however, further comparative studies are needed to standardise protocols.

## 1. Introduction

In recent years, there has been a growing interest in non-invasive skin resurfacing treatments, driven both by the increased popularity of laser technologies and by rising aesthetic expectations [1]. Fractional ablative CO_2_ lasers have long been considered the gold standard for the treatment of wrinkles, scars, and photodamage, due to their ability to generate thousands of micro-ablative zones (MAZs) and micro-thermal zones (MTZs) of injury [2,3]. This leads to a faster healing process and a lower risk of side effects compared with standard resurfacing achieved with continuous ablation [4,5,6]. To enhance collagen stimulation, fractional CO_2_ lasers have also been combined in some devices with two bipolar electrodes that emit radiofrequency (RF) [7,8]. However, the use of this technology may occasionally lead to side effects such as prolonged erythema due to the reduced selectivity of RF for water [9,10]. Several strategies have been proposed to overcome these limitations and improve the clinical outcomes without increasing tissue damage [11,12,13].

Among these, the sequential combination of a CO_2_ laser with non-ablative wavelengths, such as 1540 or 1570 nm, has attracted increasing interest [14]. These sources, belonging to the near-infrared spectrum, act on water and induce a sub-ablative thermal stimulus at the dermal level, promoting neocollagenesis without damaging the epidermis [15,16,17,18]. The aim is to achieve a synergistic thermal gradient that may improve treatment efficacy while reducing downtime [19]. Early preclinical and clinical data suggest promising results regarding scar modulation and skin rejuvenation [20,21,22].

The aim of this review is to provide a comprehensive overview of the preclinical and clinical evidence on the combined and sequential use of fractional CO_2_ laser with either 1540 nm or 1570 nm wavelengths. Current applications, histological and cellular data, clinical protocols, and the main knowledge gaps to be addressed by future studies will be analysed, with particular focus on the comparative performance, tolerability, and flexibility of these systems. To identify relevant literature, we performed a targeted search on PubMed, including all available studies assessing the use of fractional CO_2_ lasers in combination with either 1540 nm or 1570 nm wavelengths for dermatological purposes. The search strategy included keyword combinations such as “fractional CO_2_ laser AND 1540”, “fractional CO_2_ laser AND 1570”, “10600 AND 1540”, “10600 AND 1570”, and related terms.

## 2. Preclinical Background

Preclinical studies conducted at both histological and biological levels have demonstrated the synergistic effect of combining a fractional CO_2_ laser with a non-ablative wavelength, such as 1540 or 1570 nm [23,24,25].

Nisticò et al. conducted an ex vivo study on excised ovine skin, comparing fractional CO_2_ laser alone with sequential irradiation using CO_2_ and 1540 nm laser. The addition of the 1540 nm wavelength did not increase the depth of ablation, but it significantly broadened the thermal coagulation zone surrounding each ablation column compared to CO_2_ monotherapy. The authors concluded that the combined modality enhanced all the clinical benefits of CO_2_ resurfacing, including tissue tightening and neocollagenesis [23].

The sequence of laser delivery is also crucial. The greatest thermal coverage was observed when CO_2_ preceded the 1540 nm laser. This suggests that the initial ablation facilitates deeper dermal heat dispersion by the subsequent non-ablative wavelength [23].

In vivo animal studies confirmed the efficacy and safety of dual-wavelength approaches [24].

Snast et al. performed a controlled trial on porcine skin, comparing fractional CO_2_, 1570 nm, and sequential CO_2_ + 1570 nm treatments at various energy settings. Histological analysis showed that the depth of ablation was similar between CO_2_ monotherapy and dual-laser treatment at matched CO_2_ energy levels, indicating no increase in vertical tissue injury. However, consistent with the ex vivo findings, the coagulation zones were significantly broader in the CO_2_ + 1570 nm group, suggesting a wider area of controlled dermal heating. Synergistic toxicity was not observed. Healing kinetics and inflammatory profiles were comparable between the treatment groups. The authors concluded that the dual-wavelength protocol was well tolerated and histologically equivalent to standard fractional lasers in terms of the acute repair response [24].

The regenerative potential of the dual-laser approach was further supported by the findings of cellular studies. Magni et al. studied how a 1540 nm fractional laser impacted human dermal fibroblasts in culture. They found that it boosted collagen production genes and increased mitochondrial activity, while significantly enhancing type III collagen expression [25]. These results suggest that non-ablative 1540 nm laser can stimulate neocollagenesis, contributing to the overall remodelling effects when used in combination with ablative lasers. These results, summarised in Table 1, support clinical research evaluating sequential CO_2_ and 1540/1570 nm laser protocols in tissue remodelling.

## 3. Clinical Evidence

Accumulating clinical evidence over the past few years has begun to validate the anticipated benefits of sequential CO_2_ + 1540/1570 nm laser treatments. Both prospective studies and retrospective case series have explored this dual-laser approach for a range of indications, including atrophic acne scars, hypertrophic burn scars, striae distensae, and hidradenitis suppurativa, as well as for skin tightening and wrinkle reduction on the face and neck. Below, we review the evidence based on clinical indications. The key outcomes are summarised in Table 2, which also includes the level of evidence categorised as high, medium, or low. High-level evidence refers to randomised controlled trials or large prospective studies; medium refers to prospective or retrospective case series with more than 10 patients; and low refers to studies with five or fewer patients. Since no randomised controlled trials or large prospective studies were identified, only medium and low levels are reported. Studies for which the full text was unavailable were excluded from the table.

### 3.1. Atrophic Acne Scars

Treatment of atrophic acne scars is a difficult challenge that usually requires fully ablative lasers or deep chemical peels [37,38,39]. Fractional CO_2_ lasers have become a standard treatment for acne scars because they can produce substantial improvements in scar depth and texture [40,41,42].

The use of non-ablative lasers could provide additional benefits for collagen stimulation in acne-scarred dermal tissues [43,44].

Naranjo García et al. conducted a prospective study on 16 adult patients with atrophic acne scars and phototype II–IV, treated with a multimodal laser combining CO_2_ fractional ablation and non-ablative 1570 nm emission in sequential mode (Alma Lasers Ltd., Caesarea, Israel). Each patient received three sessions at two-month intervals. The treatment was tailored to the aesthetic subunit as follows: type of scar and skin phototype. Six months after the third session, the three-dimensional analysis of the skin surface showed an average reduction in scar volume from 5.7 ± 5.2 mm^3^ to 3.1 ± 3.0 mm^3^. The affected surface area also improved significantly (−43.2 ± 8.6%). Patients reported high aesthetic satisfaction, and no serious adverse events were observed. These data suggest that the sequential CO_2_ + 1570 nm approach can significantly improve atrophic acne scars, with good tolerability and a favourable safety profile [36].

Belletti et al. reported one of the first clinical pilots of a sequential 1540 nm + CO_2_ laser for atrophic acne scars. In this communication, four adult patients with moderate-to-severe facial atrophic acne scars underwent 2–4 sessions of combined 1540 nm and 10,600 nm fractional treatment, spaced 6–12 weeks apart. The dual-laser approach was delivered using a scanning system that applied both wavelengths sequentially in each pulse (DuoGlide, DEKA M.E.L.A Srl, Florence, Italy). All the patients showed noticeable scar improvement after treatment. Three months after the final session, 75% of the patients rated their acne scars as having a good to excellent improvement, while the remaining 25% noted a slight improvement. Importantly, downtime and side effects were limited. The average post-procedure downtime was about 5–6 days [26].

The authors highlighted that these results appeared comparable to those achieved with conventional fractional CO_2_ resurfacing for acne scars, but with a lower risk of complications and faster recovery [26].

Early evidence suggests this dual approach can safely deliver outcomes comparable with aggressive CO_2_ laser resurfacing for acne scars, while minimising risks such as scarring or dyspigmentation.

### 3.2. Hypertrophic Scars and Keloids

Hypertrophic scars and keloids are characterised by excessive collagen deposition, which leads to raised and firm lesions. Different laser types can be used to treat these challenging conditions [45,46]. Ablative fractional lasers can soften and flatten hypertrophic scar tissue by partially ablating and coagulating the fibrotic dermis, promoting remodelling [47,48,49]. Non-ablative lasers alone have also shown improvements in scar texture and flexibility [50,51].

The combination of CO_2_ with 1540/1570 nm represents a promising modality for scar treatment, aiming to remodel scar tissue while minimising patient downtime.

Campolmi et al. described a notable case report of a severe hypertrophic burn scar on the foot of a 34-year-old woman, treated with a multimodal laser approach, including sequential CO_2_ + 1540 nm sessions. The patient had a large, longstanding hypertrophic scar, which caused pain and restricted mobility. Initial therapy consisted of two fractional CO_2_ laser sessions, spaced one month apart (using a deeper ablation setting), to ablatively reduce the thick scar tissue. Then, three monthly sessions of combined fractional CO_2_ + 1540 nm were applied using the µScan DOT scanner (DuoGlide, DEKA M.E.L.A Srl, Florence, Italy) to further improve scar pliability and elasticity. This regimen produced a remarkable clinical response. The hypertrophic burn scar became softer, flatter, and more flexible, with a noticeable reduction in scar size. The patient experienced relief from walking pain and was able to comfortably wear regular footwear. The authors attributed the success in part to the dual-laser’s volumetric thermal effects, causing contraction and stimulation of the scar tissue, which enhanced laxity and remodelling beyond what CO_2_ alone achieved [27].

Fiorentini et al. reported a case of a post-surgical hypertrophic scar located in the suprapubic area, treated with sequential synergistic emission of fractional CO_2_ and 1540 nm laser (DuoGlide, DEKA M.E.L.A Srl, Florence, Italy). The patient presented with a scar resulting from abdominoplasty, which was symptomatic and clinically relevant in terms of thickness and discoloration. The treatment protocol involved three sessions at an interval of about 50 days, using a single, combined pass over the entire scar area. After six months of follow-up, a clear clinical improvement was observed, particularly in scar texture. No adverse events were reported, and the procedure was well tolerated. The authors highlighted that the sequential emission of the two wavelengths contributed to optimising both dermal remodelling and local tolerability [33].

Menashe et al. conducted a larger clinical study to evaluate outcomes in hypertrophic and keloid scars using a hybrid CO_2_ + 1570 nm laser (Alma Lasers Ltd., Caesarea, Israel). In this retrospective analysis, 61 scars (31 hypertrophic scars and 30 keloids) in various anatomical locations were treated with the dual-laser approach in Hybrid ProScan mode, typically using a 1:1 ratio of CO_2_ and 1570 nm energy in each pulse. The patients received a series of treatments (often 2–4 sessions) and were followed up for six months. The Vancouver Scar Scale (VSS), a clinician-rated scar severity score, showed significant improvements. This corresponds to substantial flattening, improved texture, and colour normalisation in both the scar types. In direct comparisons, hypertrophic scars responded slightly better than keloids, which was expected because keloids are biologically more aggressive. The authors concluded that the combined CO_2_ + 1570 nm device was safe and effective, with promising results for the treatment of hypertrophic and keloid scars [52].

This study is one of the first larger studies to support the use of dual-laser systems in scars. This finding suggests that the synergy between ablation and thermal stimulation can modulate pathological collagen deposition and lead to scar maturation.

In clinical practice, this may offer an evolution of the previous fractional CO_2_ lasers devices that can be combined with therapies such as dye lasers, surgery, or corticosteroid injections for challenging scars [53,54].

### 3.3. Striae Distensae

Striae distensae, or stretch marks, represent an atrophic dermal condition characterised by the degeneration of elastic and collagen fibres within the reticular dermis. Although not clinically harmful, their aesthetic and psychological impact is often considerable, particularly in the abdominal, gluteal, and femoral regions [55]. Various treatment modalities have been proposed over the years, although no consensus currently exists regarding the most effective approach [56]. Among these, fractional laser treatment, both ablative and non-ablative, has shown promising results in promoting dermal regeneration and improving the appearance of these lesions [57,58].

Menashe et al. conducted a retrospective study involving 28 patients with striae distensae, treated using a hybrid dual-wavelength laser (Alma Lasers Ltd., Caesarea, Israel). Treatments consisted of the simultaneous emission of ablative and non-ablative pulses, with parameters adjusted according to the anatomical site and skin phototype. Efficacy was evaluated using the Global Aesthetic Improvement Scale (GAIS) and a patient-reported satisfaction score on a 0–10 scale. The mean GAIS score was 7.36 ± 1.06, with a positive correlation observed between subjective satisfaction and aesthetic outcome (r = 0.685). No relevant adverse events were reported. The authors concluded that the combined use of ablative and non-ablative wavelengths provides a regenerative synergy, capable of achieving visible aesthetic improvement with reduced procedural downtime [59].

### 3.4. Neck Skin Laxity

Skin laxity of the neck is a common aesthetic concern, but it can be challenging to treat with lasers because the neck skin is thinner, more prone to scarring, and often has a high baseline risk of post-inflammatory hyperpigmentation [60]. Traditional aggressive CO_2_ resurfacing is usually avoided on the neck for these reasons. The dual-wavelength fractional laser, providing gentler overall treatment for equivalent effects, has recently been tried on the neck with positive outcomes.

Belletti et al. reported a pilot treatment of age-related neck skin laxity in two elderly female patients using a sequential CO_2_ + 1540 nm laser approach. Both patients (ages 72 and 73 years) had visibly lax, wrinkled neck skin (Fitzpatrick III) and desired non-surgical tightening. They underwent three sessions of combined 10,600/1540 nm fractional laser (DuoGlide, DEKA M.E.L.A Srl, Florence, Italy) at 45-day intervals. Three months after the final session, clinical evaluation showed a clear improvement in neck tightness and skin texture, with a reduction in the severity of horizontal neck rhytids. Using the Fitzpatrick Wrinkle Classification Score (FWCS) as an objective measure, one patient’s neck wrinkle score dropped from 8 (deep, numerous wrinkles) to 4, and the other from 6 to 3, indicating an approximate 50% improvement in wrinkle depth and severity. Patients themselves rated the results as “excellent” on a 4-point Global Aesthetic Improvement Scale and noted that their neck appeared 6–7 years younger than before. Transient erythema was expected; however, no persistent redness, scarring, or pigmentary changes were observed [28].

These findings are remarkable given that aggressive laser treatment of the neck often carries a high risk of complications. It appears that the dual-wavelength fractional approach allowed for effective collagen remodelling in the neck with a controlled, fractional injury from which the skin could heal readily. The authors cite this as evidence that the CO_2_ + 1540 nm combination can be a safe, non-surgical option for skin tightening in delicate areas such as the neck [28].

While the sample size was very small (two cases), these results open the door to further research on dual-laser neck rejuvenation in larger cohorts. This technique could represent a consistent upgrade from the use of only a CO_2_ laser for mild to moderate laxity, representing a valid alternative to radiofrequency-based devices.

### 3.5. Facial Wrinkles and Skin Rejuvenation

The most widespread application of fractional lasers is facial skin rejuvenation, which improves fine lines, wrinkles, and the overall texture and tone. Ablative fractional CO_2_ laser resurfacing is well known to induce significant improvement in facial wrinkles and rhytides, at the cost of post-treatment downtime (often 1–2 weeks of healing and redness) [61,62]. In contrast, non-ablative lasers produce a more modest wrinkle reduction but with only a few days of mild redness [63].

Hybrid lasers could be particularly useful for facial resurfacing, achieving a better efficacy to downtime ratio.

Belletti et al. conducted a prospective study on full-face rejuvenation and wrinkle reduction using a combined CO_2_ + 1540 nm laser in 20 female patients. Treatments were performed with 2–4 sessions per patient, spaced 6–12 weeks apart, using the µScan DOT scanner (DuoGlide, DEKA M.E.L.A Srl, Florence, Italy) to deliver 1540/10,600 nm sequentially in each scanned spot. Efficacy was evaluated three months after the final session using the Fitzpatrick Wrinkle Classification Scale (FWCS) and global assessments. The results demonstrated a significant improvement in wrinkles: the mean FWCS score dropped from 5.45 at baseline to 3.30 after treatment (a 39% reduction, *p* < 0.01). In practical terms, this means that patients went from having moderate wrinkles to having only mild fine lines on average. No severe adverse events were reported. The average downtime (healing time) was on the order of 5–7 days, shorter than typically seen with deep fractional CO_2_ resurfacing [29].

The authors concluded that the dual-wavelength technique may become a promising new option for safe, non-surgical improvement for skin rejuvenation with an extremely low risk of scarring or hypopigmentation and shorter healing times [29].

Shenhav et al. reported a retrospective study on a hybrid CO_2_ + 1570 nm laser (Alma Lasers Ltd., Caesarea, Israel) for periorbital rejuvenation. Shenhav’s group addressed the question of single aggressive treatment vs. multiple low-dose treatments with the hybrid laser in facial resurfacing [31].

Patients were divided into two groups: Group A received a single, high-energy CO_2_ + 1570 nm treatment, while Group B received 2–3 sessions at lower energy. The outcomes showed that while both approaches achieved comparable overall improvements (around 51–75% by GAIS), the multiple-treatment group had clear advantages such as higher satisfaction and a shorter average downtime (4.3 ± 1.6 days) compared to group A (7.3 ± 2.3 days). These findings align with fractional laser principles: splitting the total energy into a few sessions allows cumulative benefits with less risk at each visit [31].

Mezzana et al. evaluated the clinical efficacy of a simultaneous dual-wavelength emission (YouLaser MT, QuantaSystem SPA, Samarate, Italy), combining fractional CO_2_ (10,600 nm) and GaAs (1540 nm) lasers for facial photorejuvenation. In this prospective, controlled study, twenty female patients with moderate photodamage were divided into two homogeneous groups. Group A received a single pass with fractional CO_2_ laser, while group B was treated with simultaneous CO_2_ + 1540 nm emission in mixed mode. All treatments were performed with the same energy parameters and the same scanning system. Wrinkles and pigmentation were quantified objectively by three-dimensional imaging (Antera 3D), before treatment and at a three-month follow-up. Group B showed significantly better results in the reduction in fine wrinkles (32.3% vs. 20.7%, *p* = 0.0081), recovery time (3.7 vs. 5.9 days, *p* = 0.0003), and patient-reported satisfaction (mean score 3.8 vs. 2.7, *p* = 0.0032). Post-treatment erythema resolved more rapidly in the hybrid group and no significant adverse events were observed. The authors concluded that simultaneous dual-wavelength fractional resurfacing could offer a better balance between efficacy and tolerability than CO_2_ monotherapy [32].

Bonan et al. evaluated a CO_2_ + 1540 nm laser-assisted technique, specifically applied to the upper and lower eyelids in 38 patients. The dual-wavelength procedure was assessed using standardised photographs and a blinded evaluator, who scored aesthetic outcomes in four categories. At a six-month follow-up, 84% of patients showed marked improvement (75–100%), while 11% showed moderate and 5% slight improvement. No patients exhibited poor or absent response. Importantly, no serious adverse events were observed. These results support the application of dual sequential emission also in delicate areas such as the periocular region, with high aesthetic efficacy and reduced risk profile [35].

In summary, clinical evidence in the realm of facial rejuvenation consistently indicates that sequential CO_2_ + 1540/1570 nm fractional lasers can achieve significant wrinkle reduction and skin tightening.

The dual-wavelength fractional approach is emerging as an excellent option for patients seeking substantial skin rejuvenation without the extended recovery of fully ablative resurfacing.

### 3.6. Hidradenitis Suppurativa

Hidradenitis suppurativa (HS) is a chronic inflammatory disease involving the pilosebaceous units of the intertriginous areas, with a relapsing course and a significant impact on quality of life. In moderate to severe cases, treatment is often complex, and the available treatment options show variable efficacy and high recurrence rates [64].

The use of laser systems has been progressively introduced as a strategy to reduce local inflammation, remove involved follicular units and promote dermal remodelling [65].

Lindén et al. reported a case series involving eight patients with Hurley stage II-III HS, treated with dual-wavelength hybrid laser technology (YouLaser MT, Quanta System), combining fractional CO_2_ (10,600 nm) and Ga-As (1540 nm) emissions. Patients received between 3 and 26 sessions, with parameters adjusted according to skin phototype and clinical response. In all cases, lesion activity was reduced, local inflammation decreased, and skin mobility in scar areas improved. Complete resolution of active lesions occurred in some patients, along with a reduced frequency of flare-ups. The treatment was well tolerated, and no significant adverse events were reported.

According to the authors, the combination of selective ablation and deep thermal stimulation provided by the dual system allows the elimination of altered follicular structures and the modulation of the inflammatory dermal environment, with a potential benefit in the long-term control of the disease [34].

## 4. Comparative Analysis: CO_2_ + 1540 nm vs. CO_2_ + 1570 nm vs. CO_2_ + RF

The 1540 nm and 1570 nm wavelengths are part of the near-infrared spectrum. They target water as their chromophore for non-ablative dermal heating. In practical terms, 1540 nm lasers and 1570 nm lasers function very similarly, creating thermal injuries in the dermis without surface ablation [15]. The small difference in wavelength (30 nm) does not appear to produce a clinically meaningful difference in penetration or effect; rather, the device design and pulse parameters likely play a larger role.

In preclinical studies, both combinations achieved the fundamental goal of widening the thermal coagulation zone around the CO_2_ ablation columns. There is no direct comparative study of 1540 and 1570 nm in the same model, but each has independently demonstrated the key histological effect of synergy (Table 1). Safety profiles in preclinical models are comparable: neither the 1540 nm nor the 1570 nm combined approach caused additive damage to the surrounding tissue beyond what fractional CO_2_ alone would cause.

Clinically, when comparing outcomes across studies, the results with CO_2_ + 1540 nm and CO_2_ + 1570 nm appear similar in magnitude of improvement. While direct numerical comparison is difficult due to different scales, both wavelengths can clearly produce significant clinical improvement in scars and wrinkles when combined with CO_2_. The downtime reported was also in the same range.

Another consideration is device-specific characteristics. All three platforms are highly customizable (DuoGlide, Alma Hybrid, and YouLaser MT), allowing adjustment of energy, pulse duration, and density for both wavelengths.

Compared to fractional CO_2_ combined with RF, sequential CO_2_ + 1540/1570 nm lasers appear to provide more uniform dermal heating, precise energy delivery, and synergy within the same microthermal zone. Healing times remain comparable to those of CO_2_ monotherapy, with less oedema and erythema than RF-based protocols.

In summary, while fractional CO_2_ alone is highly effective, the dual-wavelength approach provides superior versatility. It achieves comparable results with less aggressive CO_2_ settings.

## 5. Future Perspectives and Conclusions

Sequential fractional CO_2_ combined with 1540/1570 nm lasers represents a relatively recent innovation in dermatologic laser therapy. Further research is necessary to strengthen the current evidence base and broaden clinical understanding, although preliminary outcomes are promising.

This dual-wavelength strategy requires larger, controlled studies to confirm its synergistic benefits and evaluate its potential superiority over other combination modalities such as CO_2_ lasers with radiofrequency.

Future investigations should explore its utility in both dermatologic and other relevant conditions requiring deep dermal remodelling [66,67,68].

The combined use of CO_2_ and 1540/1570 nm lasers appears to be a powerful and well-tolerated tool for physicians based on current preclinical and clinical data and early clinical experience. This approach is consistent with the evolving paradigm in aesthetic dermatology: maximising efficacy with minimal patient downtime and adverse effects.

## Figures and Tables

**Table 1:** Comparative histological and biological effects.

Author	Model	Laser Combination	Key Findings
Nisticò et al. [23]	Excised ovine skin	CO_2_ + 1540 nm	Wider coagulation halo around ablation columns; improved dermal heating
Snast et al. [24]	Porcine skin (in vivo)	CO_2_ + 1570 nm	Broader coagulation zone without increased depth; good healing profile
Magni et al. [25]	Human dermal fibroblast culture	1540 nm only	Upregulation of collagen genes; increased mitochondrial activity

**Table 2:** Clinical studies using fractional CO_2_ combined with 1540/1570 nm lasers.

Study (Indication, Skin Type, Level of Evidence)	Laser Wavelengths and Device	Laser Settings	Sessions (Interval)	Adverse Effects
Belletti et al., 2023 [26]—Acne scars (FP I–II) Low	CO_2_ + 1540 nm (DuoGlide)	CO_2_ 10–15 W, 900–1000 µs pulse (SP), spacing ~600 µm, Stack 1–2 (≈20–50 mJ); 1540 nm: 15–20 mJ/spot	2–4 sessions (6–12 weeks apart)	Mild oedema in all; moderate erythema in 3/4 patients; downtime ~5–6 days; no PIH or scarring
Campolmi et al., 2023 [27]—Burn hypertrophic scar (FP VI) Low	CO_2_ + 1540 nm (DuoGlide)	Initial 2 sessions CO_2_ -only: 25 W, 500 µm, HP mode, Stack 2; then CO_2_ ~15 W, 500 µm, HP mode (1 ms); 1540 nm: ~3 W, 5 ms, single-pass	3 combined sessions (monthly)	Moderate erythema/swelling; no infection; scar softening; no PIH
Pennati (Fiorentini) et al., 2023 [28]—Neck laxity (FP III) Low	CO_2_ + 1540 nm (DuoGlide)	CO_2_ ~26 mJ/dot; 1540 nm ~10 mJ/dot; scanner with contact sensor; single pass over entire neck	3 sessions (45-day intervals)	Mild to moderate erythema up to 1 week; no PIH, scarring, or contracture; well tolerated with topical anaesthesia
Belletti et al., 2023 [29]—Facial wrinkles (FP II–III) Medium	CO_2_ + 1540 nm (DuoGlide)	CO_2_ ~12 W average, 1 ms, 600 µm spacing (≈30 mJ); 1540 ~15 mJ; 1 pass full-face, ~15% coverage	2–4 sessions (6–12 weeks apart)	Erythema in 90% (resolved <10 days); 1 transient severe; oedema mild (75%) or moderate (25%); no infection, PIH, or scarring
Shenhav et al., 2023 [30]—Periorbital (FP II–III) Medium	CO_2_ + 1570 nm (Alma Hybrid)	CO_2_ ~30 mJ/spot; 1570 nm: ~20 mJ/spot; 1:1 Hybrid mode; 1 pass around each eye	1 session	Mild-moderate erythema/oedema; ~12% transient PIH (resolved); no scarring or infection; tolerable pain with topical anaesthesia
Shenhav et al., 2024 [31]—Full-face resurfacing (FP II–III) Medium	CO_2_ + 1570 nm (Alma Hybrid)	Group A: CO_2_ ~60 mJ, 1570 ~50 mJ (1 session, high fluence); Group B: CO_2_ ~30–40 mJ, 1570 ~20–30 mJ (2–3 sessions, 20% density)	A: 1 session B: 2–3 sessions (1 month apart)	A: downtime 7.3 ± 2.3 days, more crusting B: downtime 4.3 ± 1.6 days; milder erythema; no persistent complications in either group
Mezzana et al., 2016 [32]—Facial rejuvenation (FP II–III) Medium	CO_2_ + 1540 nm (YouLaser MT)	Group A: CO_2_ ~20 W, 1 ms, 300 μm spot; Group B: CO_2_ ~15 W, 0.5 ms + 1540 ~7 W, 4 ms	1 session	A: erythema/swelling resolved in 48 h, downtime 5.9 days B: resolved in 24 h, downtime 3.7 days. No long-term side effects in either group after 3 months
Fiorentini et al., 2023 [33]—Post-surgical scar (FP II) Low	CO_2_ + 1540 nm (DuoGlide)	CO_2_: 16 W, 1 ms pulse, stack 2, 500 µm spacing;1540 nm: 3 W, 5 ms pulse, stack 2;	3 sessions (every 50 days)	None reported
Lindén et al., 2022 [34]—Hidradenitis suppurativa (FP I–IV) Low	CO_2_ + 1540 nm (YouLaser MT)	Skin type I–III: CO_2_ ~16 W, 0.5 ms, 8 mJ; 1540 ~8 W, 6 ms, 48 mJSkin type IV–VI: CO_2_ ~16 W, 0.25 ms, 4 mJ; 1540 ~8 W, 4 ms, 32 mJ	5–33 sessions	Mild redness and burning lasting 1–2 h post-treatment; no long-term adverse effects
Bonan et al., 2023 [35]—Upper and lower eyelid rejuvenation (FP II–III) Medium	CO_2_ + 1540 nm (DuoGlide)	CO_2_ ~12 W, 500 μm spacing, 800 µs dwell time, DP pulse, stack 2; 1540 nm ~5 W, 3 ms dwell time	1 session	Mild erythema and oedema in 24% of cases, resolved within ~7 days; no serious adverse effects reported
García et al., 2024 [36]—Atrophic acne scars (FP II–IV) Medium	CO_2_ + 1570 nm (Alma Hybrid)	CO_2_ ~45 W, 1 ms; 1570 nm ~10 W, 3 ms; sequential grid mode	3 sessions (2 months apart)	Temporary erythema (1–2 days), swelling (2–4 days); no serious AEs reported

## Data Availability

Not applicable.
